# Identification of Tartary Buckwheat (*Fagopyrum tataricum* (L.) Gaertn) and Common Buckwheat (*Fagopyrum esculentum* Moench) Using Gas Chromatography–Mass Spectroscopy-Based Untargeted Metabolomics

**DOI:** 10.3390/foods12132578

**Published:** 2023-07-01

**Authors:** Yuling Wu, Zhanming Li, Hui Zhu, Run Zi, Fang Xue, Yue Yu

**Affiliations:** 1 School of Grain Science and Technology, Jiangsu University of Food Science and Technology, Zhenjiang 212100, China; 2 National University of Singapore Suzhou Research Institute, Suzhou 215127, China

**Keywords:** metabonomics, Tartary buckwheat, chemometrics, heatmap, GC-MS

## Abstract

Tartary buckwheat has attracted more attention than common buckwheat due to its unique chemical composition and higher efficacy in the prevention of various diseases. The content of flavonoids in Tartary buckwheat (*Fagopyrum tataricum* (L.) Gaertn) is higher than that in common buckwheat (*Fagopyrum esculentum* Moench). However, the processing process of Tartary buckwheat is complex, and the cost is high, which leads to the frequent phenomenon of common buckwheat counterfeiting and adulteration in Tartary buckwheat, which seriously damages the interests of consumers and disrupts the market order. In order to explore a new and simple identification method for Tartary buckwheat and common buckwheat, this article uses metabolomics technology based on GC-MS to identify Tartary buckwheat and common buckwheat. The results show that the PLS-DA model can identify Tartary buckwheat and common buckwheat, as well as Tartary buckwheat from different regions, without an over-fitting phenomenon. It was also found that ascorbate and aldarate metabolism was the main differential metabolic pathway between Tartary buckwheat and common buckwheat, as well as the amino acids biosynthesis pathway. This study provides a new attempt for the identification of Tartary buckwheat and common buckwheat for the quality control of related agricultural products.

## 1. Introduction

Buckwheat is widely planted all over the world, and its aliases are Black Wheat and Triangle Wheat [1]. Buckwheat has long been a popular food ingredient, and it has a high nutritional value. Compared with grains, such as wheat, rice, and corn, buckwheat has a high protein content and is easy to digest. Similarly, it is famous for its high dietary fiber content. Buckwheat is also rich in minerals, especially iron, manganese, and copper. In terms of bioactive compounds, buckwheat contains flavonoids that are lacking in other grains. Flavonoids participate in many biological functions and have important health values, such as cholesterol-lowering, hypoglycemic, and antibacterial effects [2].

At present, two cultivated species are known, which are of agricultural importance; these are common buckwheat (*Fagopyrum esculentum* Moench) and Tartary buckwheat (*Fagopyrum tataricum* (L.) Gaertn). Common buckwheat is native to Southwest China and cultivated in Russia, Japan, Canada, and Europe. This species has been cultivated in Italy in a limited area in the middle and north of the Alps and Apennines. This crop is gradually being replaced by wheat and other grains with a higher yield [3]. Tartary buckwheat has a strong ecological adaptability, meaning it can adapt to environments such as shady, wet, and cold ones, and it is mostly planted in high mountain areas. Moreover, 80% of Tartary buckwheat in China is planted in the Yunnan–Guizhou–Sichuan Plateau, Qinghai–Tibet Plateau, Gansu Province, Wuling mountain area, and Qinba mountain area. It is an indispensable food crop in local people’s life. The areas where Tartary buckwheat is planted abroad are mainly distributed in Asian countries, such as Bhutan, Nepal, and India. It is also cultivated in limited areas of Slovenia, Italy, and Northern Europe.

In general, the content of flavonoids in Tartary buckwheat (40 mg/g) is higher than that in common buckwheat (10 mg/g) [4], and the content of rutin in Tartary buckwheat seeds is about 100 times higher than that in common buckwheat seeds [5]. As a kind of flavonoid, rutin has antioxidant, antihypertensive, and α-glucosidase inhibitory activities [6]. The content and composition of flavonoids in buckwheat varieties are diverse. Furthermore, the contents of 61 flavonoid metabolites and 94 non-flavonoid metabolites in Tartary buckwheat are significantly higher than those in common buckwheat (≥2 times). Non-flavonoid metabolites also give Tartary buckwheat have a higher health promotion value than common buckwheat [7].

Both types are generally mainly used as flour or coarse wheat for food consumption, so the husking and grinding processes are essential. Tartary buckwheat is thick and firm in skin, and the grinding process is more complex than that of common buckwheat. Therefore, flour and processed foods containing Tartary buckwheat are more expensive than those containing common buckwheat, resulting in food manufacturers replacing Tartary buckwheat with cheaper common buckwheat to obtain economic profits [8]. At present, Tartary buckwheat and its products are gradually receiving more public attention, and the phenomenon of common buckwheat being disguised as Tartary buckwheat commonly exists in the market. In addition, different grain flours are often used to blend into Tartary buckwheat. Among them, common buckwheat used in combination with Tartary buckwheat easily decreases its nutritional value, and even causes safety issues for patients with hyperglycemia and celiac disease [9,10]. Therefore, identifying common buckwheat from Tartary buckwheat is necessary and helpful to improve their quality control and edibility safety.

The relevant theories for distinguishing common buckwheat from Tartary buckwheat are still lacking. The existing identification methods are few and complex, mostly focusing on the identification of dietary fiber, protein, amino acid, and other nutrients or flavonoids, and include multiple real-time PCR detection methods. Metabolomics technology is simple and effective in sample processing and is flexible in data analysis. It can accurately reflect various biological functions of organisms through metabolic pathways and describe the effects of various factors on the metabolic network of organisms [11]. Therefore, it is feasible and advantageous for identifying Tartary buckwheat and common buckwheat.

At present, the techniques for studying volatile substances mainly include thin-layer chromatography (TLC), gas chromatography–mass spectroscopy (GC-MS), Fourier transform infrared spectroscopy (FTIR), two-dimensional infrared spectroscopy, and ultraviolet spectroscopy. As a mature detection method, GC-MS is widely used for the identification of volatile substances, such as honey, tea, fruit, and wine, due to its small sample size, low detection limit, and high sensitivity. GC-MS combined with orthogonal partial least-squares discriminant analysis (OPLS-DA) models showed excellent japonica rice geographic discrimination, and hexanal, 3,5-octadien-2-one, and 2-butyl-2-octenal were selected both in raw and cooked rice [12]. The untargeted metabolomics combined with partial least squares regression (PLSR) was a feasible tool used to measure the volatile metabolites in Chinese rice wine for the identification of rice age [13]. In comparison with other techniques (i.e., image processing), GC-MS combined with chemometrics is more cost-effective and easy to model.

As reported, chemometrics methods can be used for the analysis of the metabolites for a better understanding of the results. Principal component analysis (PCA), as a multivariate statistical analysis method for the dimensionality reduction in multidimensional data through data compression, possesses a simple operation, no parameter limitations, and a wide application range. It can be useful to distinguish food varieties and determine the contribution components of different varieties’ metabolite differences. Partial least squares discriminant analysis (PLS-DA), or OPLS-DA, can be used for the differential analysis of metabolites among varieties [14,15]. In comparison with other techniques (i.e., image processing), GC-MS combined with chemometrics is more cost-effectivene and easy to model.

In this study, the metabolites of common buckwheat, Sichuan Tartary buckwheat, and Yunnan Tartary buckwheat were extracted via distillation and analyzed using untargeted metabolomics based on the GC–MS technique. PCA and PLS-DA were both employed to identify the differential metabolites of common buckwheat and Tartary buckwheat. This study provides basic data and new insights for the identification and quality evaluation of common buckwheat and Tartary buckwheat.

## 2. Materials and Methods

### 2.1. Materials

Common buckwheat was obtained from various places in the Chinese provinces of Yunnan, Guizhou, Heilongjiang, and Shandong. Tartary buckwheat was purchased from different regions in the Chinese provinces of Yunnan and Sichuan. The samples were collected from the local regions during the same harvest season in 2022. Samples from different regions were collected in order to increase the quantity. Meanwhile, the samples from different regions were also involved in the identification of differences between the same variety samples, which was helpful to clarify the different properties between common buckwheat and Tartary buckwheat. Both types of buckwheat were split into groups A, B, and C, with six samples in each group (Table 1).

### 2.2. Extraction Methods

First, 50.0 g of common buckwheat and 50.00 g of Tartary buckwheat sample powder were weighed and placed in two 1000 mL flasks separately. Then, 500 mL of distilled water was measured and poured into the flasks. Glass beads were subsequently added, and the flasks were heated and slowly distilled for 4 h. Afterwards, the heat source was turned off and cooled to room temperature. The volume of volatile oil was accurately read as 0.05 mL. The distillate was continuously extracted via ethyl acetate three times and then dried via anhydrous Na_2_SO_4_ overnight. Next, the ethyl acetate was evaporated in a water bath to obtain volatile oil with a delicate fragrance [15].

### 2.3. GC-MS Analysis

An HP-5 capillary column (30 m, 0.25 mm, and 0.25 μm) with 0.1 μL sample inlet was used. The partial flow ratio was 30:1, the gasification chamber temperature was 250 °C, and the carrier gas used was high-purity He (99.999%). The temperature was programmed as follows: initial column temperature of 100 °C, ramped up at a rate of 3 °C per min^−1^ to 130 °C, held for 10 min, and then ramped up at a rate of 6 °C per min^−1^ to 250 °C [15].

For the EI ion source, the ion source temperature was 230 °C; the quad pole temperature was 150 °C, the interface temperature was 280 °C, the ionization energy was 70 eV, the EM voltage was 2165 V, the solvent delay was 3 min, and the scanning quality ranged from *m*/*z* 40 to *m*/*z* 550 [14]. The mixture of common buckwheat and Tartary buckwheat was used as QC samples (N = 3) to verify the detection performance using the same extraction and analysis methods. The NIST mass spectrometry library was used for statistical analysis. The volatile compounds were qualitatively studied by referring to the linear retention index and aroma attributes in references. Various chemical components were quantified using peak area normalization method [15,16].

### 2.4. PCA Analysis

PCA is an unsupervised data dimension reduction analysis method [17], and it is one of the widely used data analysis methods. The data obtained via GC-MS were organized and imported into SIMCA-P 14 software for statistical analysis, with PCA as the first step, to provide an overview of all observations and samples and identify and evaluate groupings, trends, and strong outliers for the intuitive analysis of the classification of common buckwheat and Tartary buckwheat [18].

### 2.5. PLS-DA Analysis

PLS-DA was applied to classify common buckwheat and Tartary buckwheat to obtain different components [15]. PLS-DA is a supervised multivariate statistical analysis method used for discriminant analysis. A linear regression model is found by projecting the predictor and the observed variables into a new space. As a supervised modeling method, the prediction of the sample category is realized by establishing the relationship model between the omics data and the sample category. Variable importance for the projection (VIP) was calculated to measure the influence of the expression mode of each indicator on the classification and discrimination of samples and the explanatory ability. VIP value >1.0 was selected in this study as an important indicator [14,19].

### 2.6. Volcano Diagram Analysis

Volcanic maps were established for the analysis of different metabolites [20,21]. Volcanic maps allow for the visual analysis of differential metabolites (up-regulated or down-regulated) in common buckwheat and Tartary buckwheat samples. The y-coordinate is |log_2_(fold change)|, and the x-coordinate is −log_10_(*p*-value). Moreover, red dots are the significantly up-regulated metabolites and green dots are down-regulated metabolites. Black spots are the metabolites with no significant difference.

### 2.7. Heatmap Analysis

Heatmap is used to visualize the data with color via a logarithmic mapping of each value in the data matrix. This method can display the changes of multiple metabolites in common buckwheat and Tartary buckwheat samples, and also show the clustering relationships of metabolites. Generally, the redder color represents the higher value. Different heatmaps (pairwise comparison) between the common buckwheat and Tartary buckwheat samples and different Tartary buckwheat samples were prepared in this study.

### 2.8. Differential Metabolite Annotation and Pathway Analysis

The HMDB database, Lipidmaps database, and KEGG database were used to annotate differential metabolites. The HMDB database contains detailed information about the human metabolome. It is the largest and most comprehensive bio-specific metabolome database in the world. It contains detailed information on small molecule metabolites in the human body and their biological effects, disease associations, physiological concentrations, chemical reactions, and metabolic pathways [22]. Lipidmaps is the largest public lipid database, including molecular formulas of lipid elements and their lipid classes [23], and it can annotate eight major lipid classes and their subclasses [24,25]. KEGG is a comprehensive database comprising multiple databases [26], among which the KEGG pathway database is a collection of metabolic pathways. It divides biological metabolic pathways into the following seven categories: metabolism, environmental information processing, genetic information processing, organismal systems, cellular processes, human diseases, and drug development [27].

## 3. Results and Discussion

### 3.1. PCA

**Figure 1:**
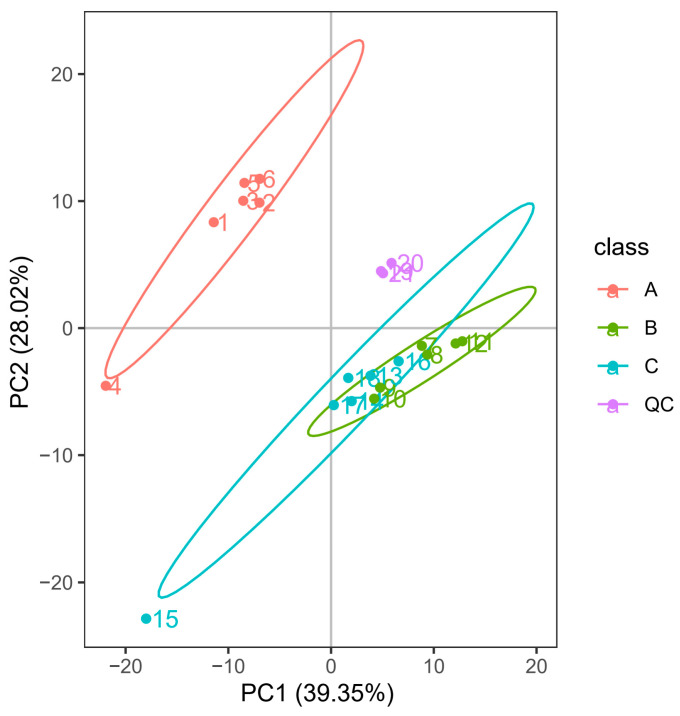
PCA results of A, B, C and QC samples. A—common buckwheat, B—Tartary buckwheat in Sichuan, C—Tartary buckwheat in Yunnan.

PCA is an unsupervised data dimension reduction analysis method [18], and it is one of the widely used data analysis methods. In the present study, PCA was performed on the differential metabolites of samples a–c, and the results are shown in Figure 1. The total score of the three principal components was available for covering most of the effective information of the original data. Moreover, the samples of Tartary buckwheat and common buckwheat could be distinguished. The QC samples presented a high correlation, indicating that the stability of the detection system was excellent, and the system is reliable for obtaining results of the generated data. However, the distribution areas of the Tartary buckwheat samples from different regions overlapped, making it difficult to distinguish them well. Therefore, a more prominent method should be used for analyzing and identifying intergroup differences.

**Figure 2:**
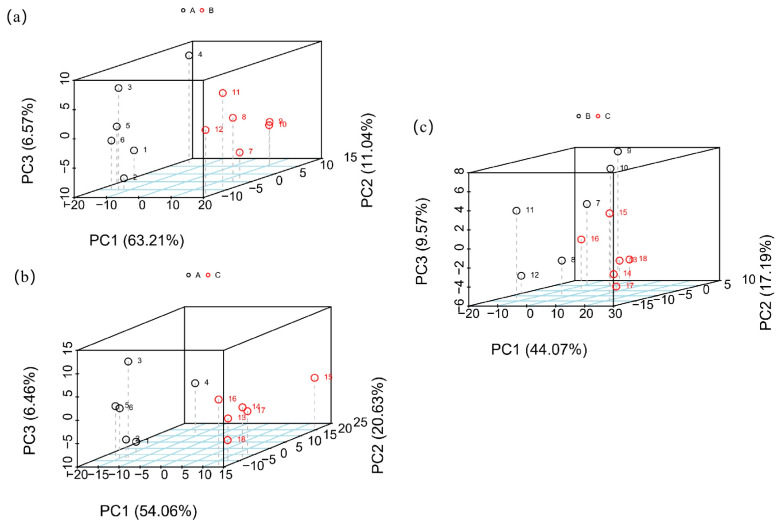
PCA results of (**a**) A versus B, (**b**) A versus C, and (**c**) B versus C. A—common buckwheat, B—Tartary buckwheat in Sichuan, C—Tartary buckwheat in Yunnan.

The PCA model was used to analyze samples A and B, A and C, and B and C, and the results are shown in Figure 2. The total contribution rates of the principal components shown in Figure 2a are all greater than 80, and the total contribution rate in Figure 2c is greater than 70. The cumulative contribution rate of the three graphs is relatively high, and the analysis results are more reliable. As shown in Figure 2a,b, the sample points of common buckwheat and Tartary buckwheat are clustered in the same area, and the aggregation areas of the different sample points differ, indicating that the PCA model can be used to distinguish samples of common buckwheat and Tartary buckwheat. In the analysis of Tartary buckwheat samples from different producing areas (Figure 2c), the distribution of sample points in the same group is relatively scattered, and the distribution of sample points in the different groups overlap, making the discrimination effect non-satisfactory. The results show that the PCA model can be used to identify common buckwheat and Tartary buckwheat, but identifying Tartary buckwheat from different areas is difficult. In previous research, metabolomics was also used to discriminate four rice samples from different regions without excellent performance [14]. More models, like the PLS-DA model, should be employed to show the discrimination accuracy for satisfactory performance.

### 3.2. PLS-DA Analysis

**Figure 3:**
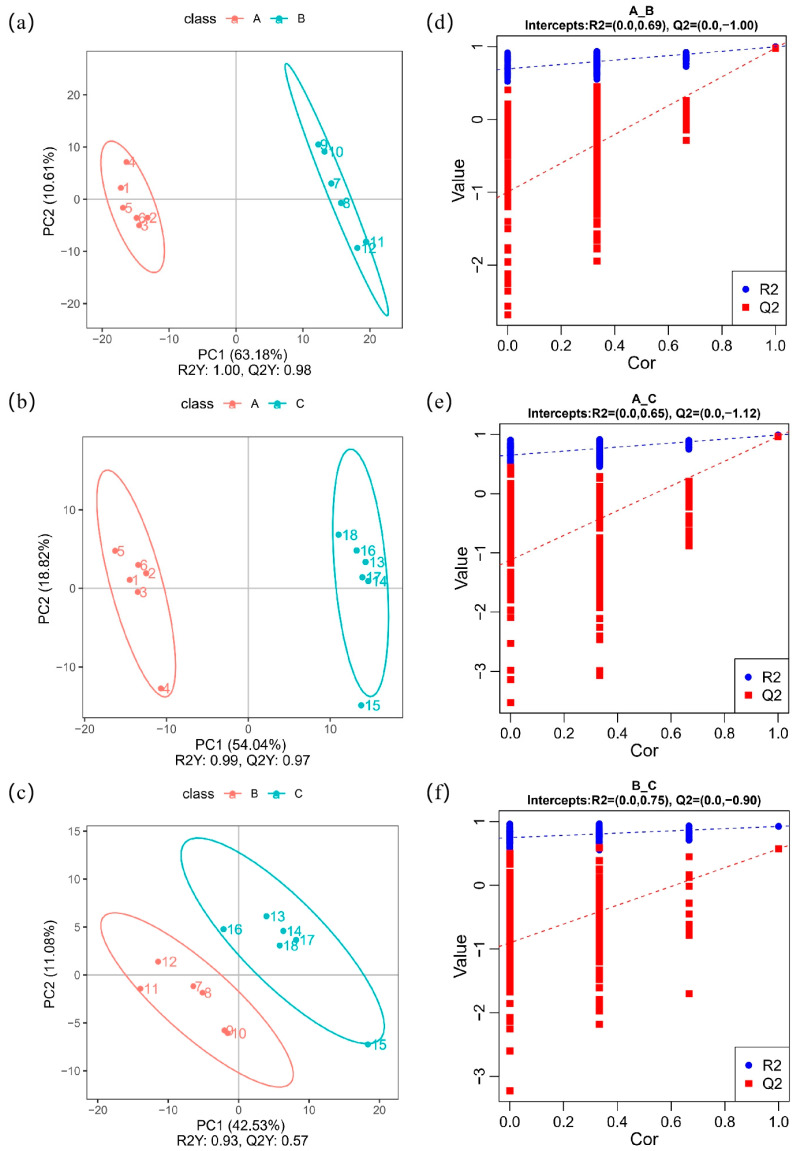
PLS-DA results of (**a**) A versus B, (**b**) A versus C, and (**c**) B versus C; permutation test of PLS-DA model: (**d**) A versus B, (**e**) A versus C, and (**f**) B versus C. A—common buckwheat, B—Tartary buckwheat in Sichuan, C—Tartary buckwheat in Yunnan.

Compared with PCA, as a supervised multivariate statistical analysis method [19], PLS-DA could further enhance the difference between the groups. In the present study, the PLS-DA model was used to analyze the differential metabolites of common buckwheat, Tartary buckwheat, and Tartary buckwheat from different regions (Figure 3a–c). R2X and R2Y represent the percentage of the X and Y matrix information that the PLS-DA model can explain, respectively. The R2Y of each PLS-DA graph is greater than 0.9, indicating that the model retained more original information, and the results are accurate. The Q2Y of Figure 3a–c is greater than 0.5, indicating that the model had a good predictive ability. In the PLS-DA model, common buckwheat and Tartary buckwheat are located on the left and right sides of the map, far away from each other, and the distribution between the same sample is compact. The distribution area of the different samples obviously differ, and the distribution of the Tartary buckwheat samples from different producing areas exhibit a certain boundary. Therefore, the PLS-DA model can be used to distinguish Tartary buckwheat, common buckwheat, and Tartary buckwheat from different producing areas.

A permutation test was carried out to verify whether the PLS-DA model was overfitted. Overfitting indicates that the results are not accurate, and that the model is not suitable for subsequent experimental analysis. The order of variable Y was randomly changed, and the corresponding PLS-DA model was re-established to obtain new R2 and Q2. The results are shown in Figure 3d–f. The Q2 in the map is less than R2, and the intercept of Q2 on the Y axis is negative, indicating that the PLS-DA model was not overfitted, and the results are accurate.

Although the classification accuracy for common buckwheat and Tartary buckwheat has reached 100%, the real challenge arises when the classification needs to be performed in more complex scenarios, such as the identification of adulteration with different ratios of common buckwheat and Tartary buckwheat. As reported, in complex scenarios of aged rice adulteration with different ratios, partial least squares regression (PLSR) and support vector machine regression (SVR) combined with spectral data preprocessing and characteristic wavelength variable screening were employed to achieve the identification [28], indicating that complex models should be developed for complex samples.

### 3.3. Volcano Diagram Analysis

The number of differential metabolites between the different samples was screened in accordance with VIP, fold change (FC), and *p*-value parameters. The VIP value represents the contribution of metabolites to the sample grouping [15], the FC refers to the presence of multiple differences, and the *p*-value indicates a significant difference. On the basis of the criteria of VIP > 1, FC > 2, or FC < 0.5 and a *p*-value < 0.05 [20], the differential metabolites were screened, and the results are shown in Table 2. The differential metabolite compounds in the three groups are presented in Tables S1–S3.

**Figure 4:**
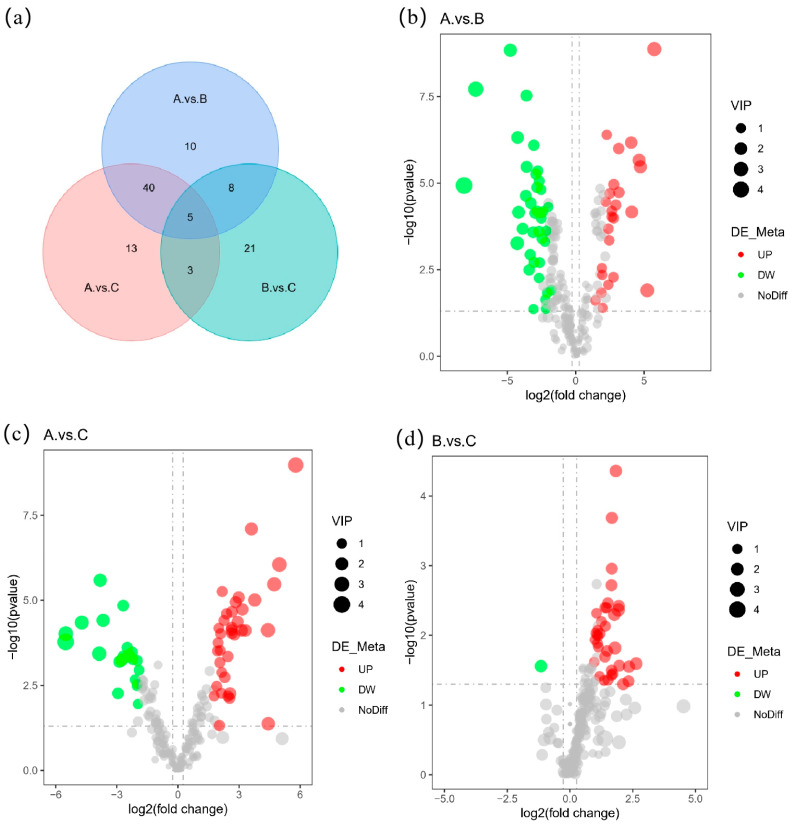
Venn analysis of differential metabolites in different groups (**a**); volcano diagram results of (**b**) A versus B, (**c**) A versus C, and (**d**) B versus C. A—common buckwheat, B—Tartary buckwheat in Sichuan, C—Tartary buckwheat in Yunnan.

The volcanic diagram (Figure 4) intuitively shows the distribution of the different metabolites between the two samples. The red point is the up-regulated point, and the green point is the down-regulated point. The abscissa is represented by log_2_(FC). The points on both sides of the distribution have larger differences, and the ordinate is represented by -log10 (*p*-value). The points with greater differences are distributed upwards [21], so the points in the upper left and upper right corners are generally more biologically significant. The figure clearly shows that compared with the common buckwheat, the number of differential metabolites in the Tartary buckwheat from different regions was significantly reduced.

### 3.4. Heatmap Analysis

**Figure 5:**
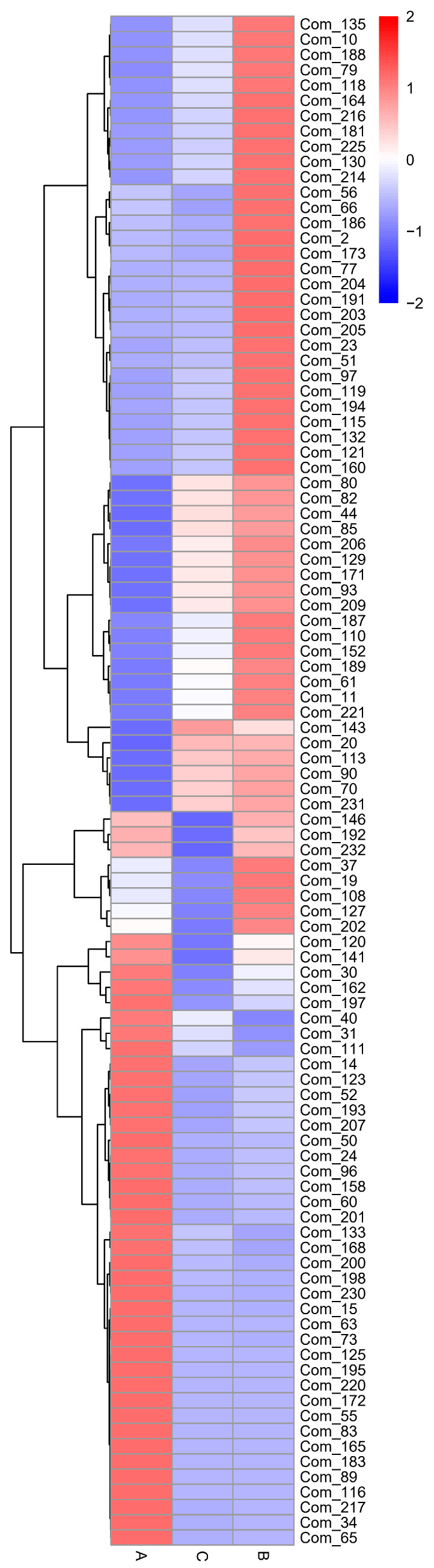
Heatmap diagram analysis of the three groups. A—common buckwheat, B—Tartary buckwheat in Sichuan, C—Tartary buckwheat in Yunnan. The metabolites’ information is shown in Tables S1–S3.

The various metabolites of the three groups of buckwheat samples are presented by heatmap, a popular data visualization method that uses color to map data for the data to become more intuitive and for the contrast to become more obvious. The rows and columns were reordered according to the relationship of the data expression, so that the differential metabolites with similar expression levels could be tightly grouped, and the global expression levels of multiple differential metabolites among multiple samples could be visually presented. In the clustering heatmap, vertical represents the grouping of samples, and level represents the clustering of metabolites; the briefer the cluster branch, the greater the similarity. The color in the heatmap changes from blue to red, where red indicates that the content of metabolites is high, and blue indicates that the content of metabolites is low. Figure 5 shows that different sample groups demonstrated different expressions of differential metabolites through the display of color. The comparison between the samples in group A and the samples in groups B and C could be used to identify common buckwheat and Tartary buckwheat, and the comparison of the samples in groups B and C could be used to identify the habitat of Tartary buckwheat. The tree-like branches at the top of the heatmap also indicate information about the similarities and differences among the samples.

The content of metabolites, such as 2,4-diaminobutyric acid 1, methyl phosphate, creatine degr, and epicatechin, in the group A samples was much higher than that in the Tartary buckwheat samples, and the difference in distribution was obvious (red or blue color at the bottom position). In the Tartary buckwheat samples of group B, the contents of galactonic acid, dehydroshikimic acid 1, isoleucine, L-allothreonine 1, and other metabolites were well distributed and significantly higher than those in the common buckwheat samples (blue at the bottom and red at the top). In the Tartary buckwheat samples of group C, the ribulose-5-phosphate 1 content was substantially higher than that in the other two groups, as shown in Figure 5. These findings show that the differences among the Tartary buckwheat samples may be caused by changes or differences in the processes of cultivation, drying, picking time, and storage. The metabolomics analysis performed in this paper presents the different characteristics of common buckwheat samples and Tartary buckwheat samples by supporting the information of the related metabolites.

The correlation analysis of the differential metabolites among different groups can help us understand the expression relationship between these metabolites. If the expression trend tends to converge, it can be preliminarily judged that the differential metabolites have a functional correlation, that is, they participate in the same or synergistic biological processes. If the expression levels of two different differential metabolites increase or decrease together, they can be considered to have a positive correlation. If one of the differential metabolites increases and the expression of the other differential metabolites decreases, this can be considered as a negative correlation.

**Figure 6:**
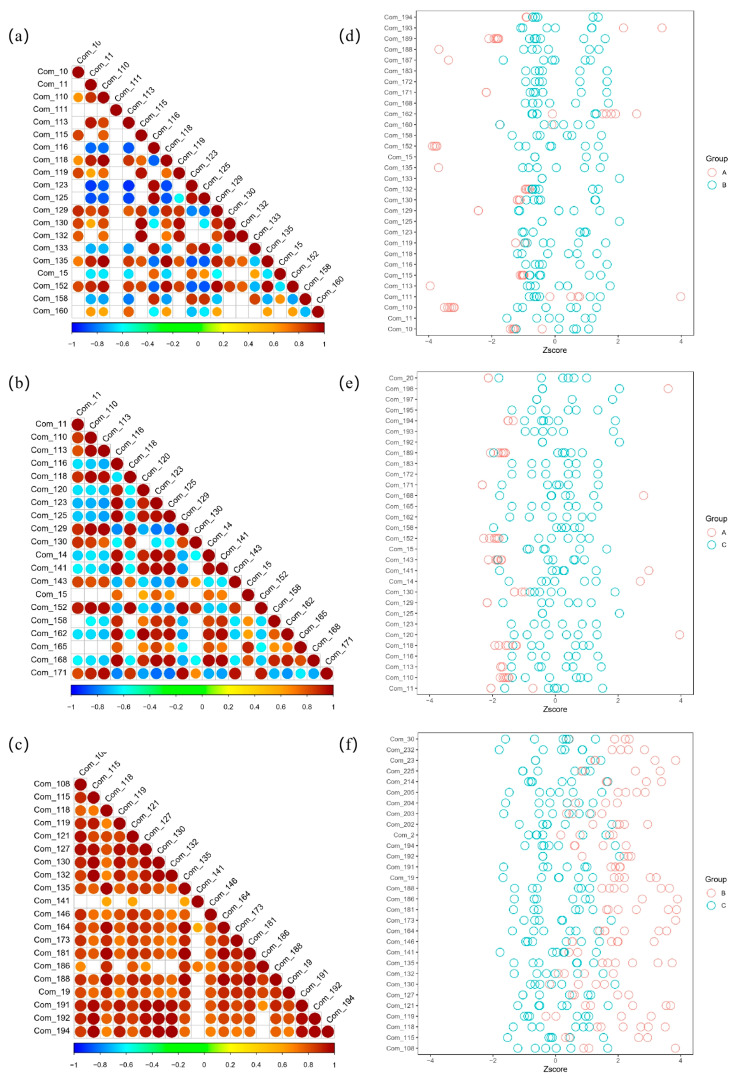
Pearson correlation analysis of (**a**) A versus B, (**b**) A versus C, and (**c**) B versus C; Z-score analysis of (**d**) A versus B, (**e**) A versus C, and (**f**) B versus C. A—common buckwheat, B—Tartary buckwheat in Sichuan, C—Tartary buckwheat in Yunnan. The metabolites’ information is shown in Tables S1–S3.

The Pearson correlation analysis was used to analyze differential metabolites (Figure 6). A positive correlation is represented by warm color dots, and the greater the correlation is, the closer it is to red. A negative correlation is represented by cool color dots, and the greater the correlation is, the closer it is to blue. According to the cool and warm colors of the three maps, the differential metabolites in the Tartary buckwheat samples from different producing areas have a strong functional correlation. In comparison with the results between the Tartary buckwheat samples, the cool color between the common buckwheat samples and Tartary buckwheat samples present a weak correlation.

As a data standardization method, the Z-score chart can eliminate the influence of dimension and order of magnitude on the data analysis, which makes it possible to compare the variables of different magnitude and dimensions vertically [29]. It can help to survey the variables to distinguish samples of different groups. As shown, the difference of the distribution between the common buckwheat and Tartary buckwheat (Figure 6d,e) is obviously different in comparison with the distribution between the Tartary buckwheat from different regions (Figure 6f).

### 3.5. Differential Metabolite Annotation and Pathway Analysis

**Figure 7:**
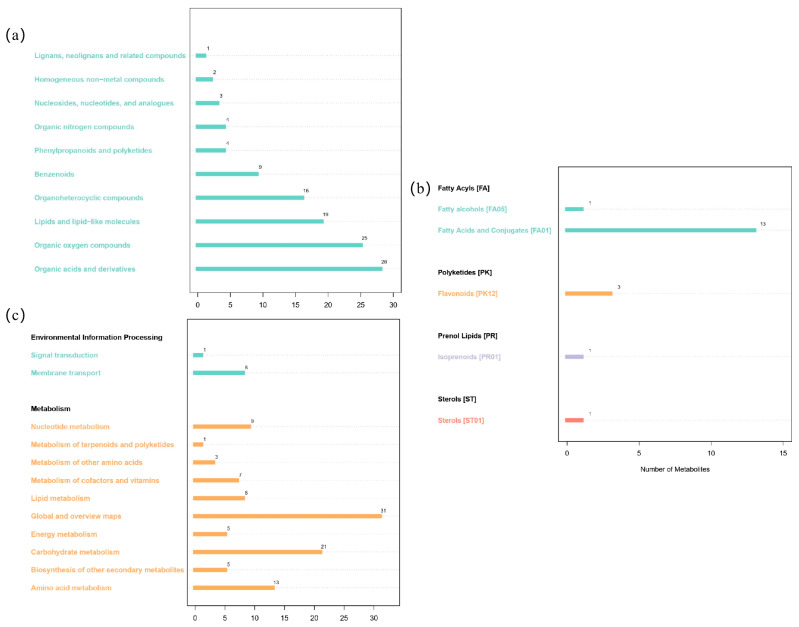
Differential metabolite annotations of (**a**) HMDB, (**b**) Lipidmaps, and (**c**) KEGG databases.

A total of 111, 19, and 112 metabolites were annotated in the HMDB database, Lipidmaps database, and KEGG database, respectively. The differential metabolites in the present study were annotated via HMDB (Figure 7a). A total of 10 categories of lignans, neolignans and related compounds, homogeneous non-metallic compounds, nucleosides, nucleotides and analogues, organic nitrogen compounds, phenylpropanoids and polyketides, benzenoids, organoheterocyclic compounds, lipids and lipid-like molecules, organic oxygen compounds, and organic acids and derivatives were found. Among them, organic oxygen compounds and organic acids and derivatives accounted for the largest proportion.

By using the Lipidmaps database for lipid analysis (Figure 7b), the differential metabolites were classified into the following four categories: fatty acyls, polyketides, prenol lipids, and sterols. Fat acyl was divided into fatty alcohols and fatty acids and conjugates, and the differential metabolites belonging to them accounted for the highest proportion. The KEGG database was used to analyze the metabolic pathways of differential metabolites in the present study. The metabolic pathways involved (Figure 7c) were environmental information processing and metabolism.

**Figure 8:**
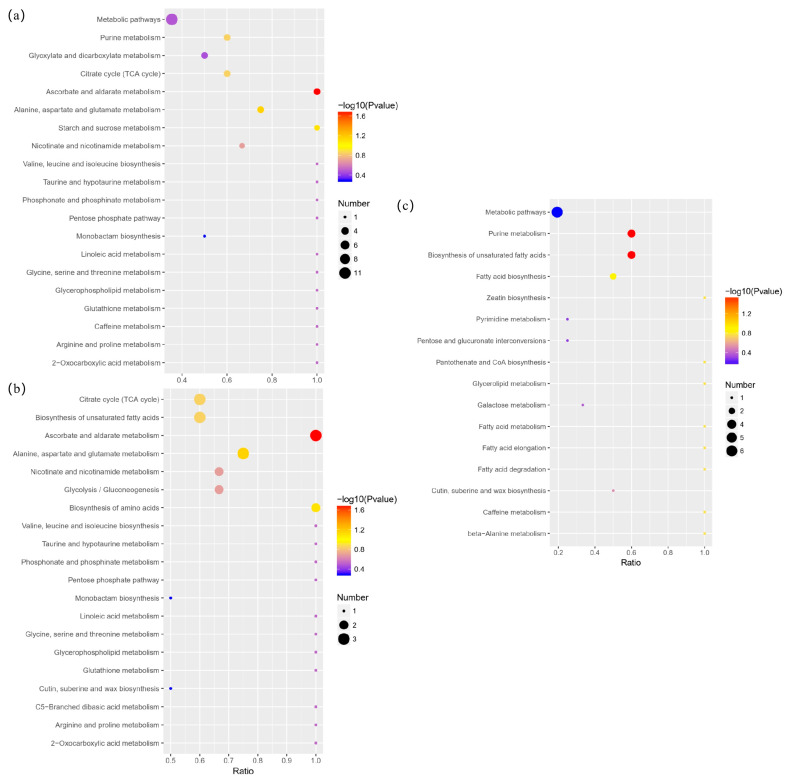
Bubble diagram of enriched KEGG pathway for (**a**) A versus B, (**b**) A versus C, and (**c**) B versus C. A—common buckwheat, B—Tartary buckwheat in Sichuan, C—Tartary buckwheat in Yunnan.

KEGG was used for the Pearson correlation analysis of differential metabolites (Figure 8), which is helpful to determine the main pathways involving differential metabolites. As shown, the ordinate is the metabolic pathway, and the abscissa is x/y (the number of differential metabolites in the corresponding metabolic pathway/the number of total metabolites identified in the pathway), indicating the enrichment of differential metabolites in the pathway. The greater the abscissa value, the higher the enrichment of the differential metabolites. The color of the point in the figure represents the *p*-value. The smaller the *p*-value is, the more the color of the point tends to be cool, indicating that the reliability of the test is greater. The size of the point represents the number of differential metabolites in the corresponding pathway. The larger the point is, the greater the number of differential metabolites in the pathway.

In the analysis map of the Tartary buckwheat and common buckwheat (Figure 8a,b), the enrichment degree of differential metabolites in ascorbate and aldarate metabolism is high. This finding shows that the ascorbate and aldarate metabolism was the main differential metabolic pathway of the Tartary buckwheat and common buckwheat. A previous study found that the proper processing treatment can effectively improve the antioxidant enzyme activity of Tartary buckwheat sprouts, which is possibly associated with the ascorbate and aldarate metabolism [30]. Drought stress decreased the growth and biomass of the Tartary buckwheat, which may also be involved in the ascorbate and aldarate metabolism [31].

For the biosynthesis of amino acids, the high enrichment degree of differential metabolites indicated that the biosynthesis of amino acids was also greatly different between the Tartary buckwheat and common buckwheat, which was similar to the findings of previous research. As reported, the changing contents of various amino acids during Tartary buckwheat seed germination can be caused by many factors [32]. It was also presented that genes involved in secondary metabolite biosynthesis including amino-acid-related genes were responsive to salt stress with a significant change [33].

As stated above, the main differential metabolic pathways clarified by the analysis of metabolites showed the different characteristics of common buckwheat samples and Tartary buckwheat samples. Untargeted metabolomics is an alternative method to determine the differences and average characteristics of common buckwheat samples and Tartary buckwheat samples using the different identified metabolites.

## 4. Conclusions

Untargeted metabolomics technology based on GC-MS was used to identify Tartary buckwheat and common buckwheat successfully. The results show that the PLS-DA model can identify Tartary buckwheat and common buckwheat without an overfitting phenomenon. Differential metabolites were obtained, and the number of differential metabolites in the Tartary buckwheat from different regions (37 compounds) was significantly smaller than that of the Tartary buckwheat and common buckwheat (63 or 61 compounds). Significant differences were presented in the metabolites among different samples using visualization analysis, including PLS-DA, volcanic map, heatmap, and Z-score. It was also found that the ascorbate and aldarate metabolism was the main differential metabolic pathway of the Tartary buckwheat and common buckwheat. The biosynthesis of amino acids was also greatly different between the Tartary buckwheat and common buckwheat. In summary, metabolomics based on GC-MS technology can be used to identify Tartary buckwheat and common buckwheat. This study provides a new method for identifying Tartary buckwheat and common buckwheat, which helps to further promote food safety.

## Figures and Tables

**Table 1:** Samples of common buckwheat and Tartary buckwheat.

Sample Number	Group Name	Buckwheat Type	Region
1	A	Common buckwheat	Kunming, Yunnan
2		Common buckwheat	Bijie, Guizhou
3		Common buckwheat	Bijie, Guizhou
4		Common buckwheat	Yilan County, Harbin, Heilongjiang
5		Common buckwheat	Zhaodong City, Suihua, Heilongjiang
6		Common buckwheat	Jining, Shandong
7	B	Tartary buckwheat	Zhaojue County, Liangshan, Sichuan
8		Tartary buckwheat	Zhaojue County, Liangshan, Sichuan
9		Tartary buckwheat	Zhaojue County, Liangshan, Sichuan
10		Tartary buckwheat	Zhaojue County, Liangshan, Sichuan
11		Tartary buckwheat	Xichang County, Liangshan, Sichuan
12		Tartary buckwheat	Xichang County, Liangshan, Sichuan
13	C	Tartary buckwheat	Qujing, Yunnan
14		Tartary buckwheat	Qujing, Yunnan
15		Tartary buckwheat	Qujing, Yunnan
16		Tartary buckwheat	Qujing, Yunnan
17		Tartary buckwheat	Zhaotong, Yunnan
18		Tartary buckwheat	Zhaotong, Yunnan

**Table 2:** Quantitative identification of differential metabolites.

Group	Number of Differential Metabolites	Up	Down
A vs. B	63	25	38
A vs. C	61	37	24
B vs. C	37	36	1

## Data Availability

The datasets generated for this study are available on request to the corresponding author.

## Supplementary Materials

The following supporting information can be downloaded at https://www.mdpi.com/article/10.3390/foods12132578/s1, Table S1: The identified differential metabolites (VIP > 1) between group A vs. group B. A-common buckwheat, B-Tartary buckwheat in Sichuan. Table S2: The identified differential metabolites (VIP > 1) between group A vs. group C. A-common buckwheat, C-Tartary buckwheat in Yunnan. Table S3: The identified differential metabolites (VIP > 1) between group B vs. group C. B-Tartary buckwheat in Sichuan, C-Tartary buckwheat in Yunnan.
