# Supplementary material for: Identification of Tartary Buckwheat (Fagopyrum tataricum (L.) Gaertn) and Common Buckwheat (Fagopyrum esculentum Moench) Using Gas Chromatography–Mass Spectroscopy-Based Untargeted Metabolomics
Source: Foods. 2023 Jul 1;12(13):2578. doi: 10.3390/foods12132578 (PMC10340619; doi:10.3390/foods12132578)
Supplement: Supplementary file 1 [file foods-12-02578-s001.zip › foods-2444438-supplementary.pdf]

Table S1. The identified differential metabolites (VIP＞1) between group A vs group B. A-common buckwheat, B-Tartary buckwheat in Sichuan.

ID	Name_des	VIP	Up.Down
Com_10	Galactonic acid	1.134928138	down
Com_11	Toluenesulfonic acid	1.434886104	down
Com_110	Sophorose 2	1.788270983	down
Com_111	Trehalose	1.032871047	up
Com_113	1,5-Anhydroglucitol	2.351090018	down
Com_115	Guanosine	1.462715857	down
Com_116	Creatine degr	1.319896379	up
Com_118	Proline	1.627096116	down
Com_119	3,6-Anhydro-d-galactose 3	1.263292805	down
Com_123	Ethanolamine	1.263325563	up
Com_125	Prostaglandin a2 3	2.82309365	up
Com_129	Kyotorphin 2	1.336597458	down
Com_130	Glucoheptonic acid 1	1.762822936	down
Com_132	Lyxose 2	1.518823637	down
Com_133	Aconitic acid	1.220003737	up
Com_135	Isoleucine	1.276439926	down
Com_15	3-(4-Hydroxyphenyl)propionic acid	2.643329123	up
Com_152	Serine 1	1.658493224	down
Com_158	Galactinol 1	1.070717352	up
Com_160	9-Fluorenone 2	1.205322829	down
Com_162	Linoleic acid	1.111839198	up
Com_168	Citric acid	1.52651288	up
Com_171	Trehalose-6-phosphate	1.20003678	down
Com_172	Hesperitin 3	1.40413406	up
Com_183	Piceatannol 2	2.284154587	up
Com_187	Hydroxyurea	1.30044429	down

Com_188	L-allothreonine 1	1.200646036	down
Com_189	Maltose	1.217830911	down
Com_193	Aspartic acid 1	1.088394593	up
Com_194	D-erythronolactone 2	4.491530251	down
Com_198	Glucose-6-phosphate 2	1.074083108	up
Com_2	D-erythro-sphingosine 2	1.016714895	down
Com_20	Ribulose-5-phosphate 1	1.339118675	down
Com_200	N-methyl-l-glutamic acid 1	1.379942007	up
Com_201	Galactose 2	1.219396381	up
Com_206	Alanine 1	1.279803891	down
Com_207	Atrazine-2-hydroxy 4	1.078579767	up
Com_209	Saccharic acid	2.604537471	down
Com_214	Methionine 1	1.735071307	down
Com_216	Uric acid	1.468383324	down
Com_217	Epicatechin	2.233971313	up
Com_220	N-methylaniline	1.143541937	up
Com_221	4-Hydroxy-3-methoxybenzoic acid	1.058907812	down
Com_225	Lactic acid	3.568139432	down
Com_231	Mucic acid	1.398689333	down
Com_31	Cytidine-monophosphate degr prod	1.217240978	up
Com_44	Oxalic acid	1.00512317	down
Com_50	Threo-beta-hydroxyaspartate 1	1.111099764	up
Com_51	Lysine	1.060717358	down
Com_55	Ascorbate	1.262097884	up
Com_61	3-Hydroxybutyric acid	1.778182573	down
Com_63	(+)-Catechin	1.961823249	up
Com_65	Methyl phosphate	2.175439946	up
Com_70	3-Hydroxypropionic acid 1	1.306348689	down
Com_79	Dehydroshikimic acid 1	1.054504	down

Com_80	4-Hydroxybutyrate	2.181218749	down
Com_82	Fucose 1	1.34632362	down
Com_83	Nicotinamide	1.520101856	up
Com_85	Pyruvic acid	2.087483519	down
Com_89	Trans,trans-muconic acid	1.261623016	up
Com_90	Guanidinosuccinic acid 3	1.163832425	down
Com_93	Succinic acid	1.510312356	down
Com_97	Xanthine	1.431432113	down

Table S2. The identified differential metabolites (VIP＞1) between group A vs group C. A-common buckwheat, C-Tartary buckwheat in Yunnan.

ID	Name_des	VIP	Up.Down
Com_11	Toluenesulfonic acid	1.154112715	down
Com_110	Sophorose 2	1.5030924	down
Com_113	1,5-Anhydroglucitol	2.55660118	down
Com_116	Creatine degr	1.524358241	up
Com_118	Proline	1.172864028	down
Com_120	Oleic acid	1.828292326	up
Com_123	Ethanolamine	2.059055742	up
Com_125	Prostaglandin A2 3	3.27179634	up
Com_129	Kyotorphin 2	1.299566681	down
Com_130	Glucoheptonic acid 1	1.197587368	down
Com_14	N-Acetyl-beta-D-mannosamine 4	1.15075927	up
Com_141	4-Acetylbutyric acid 2	1.137101167	up
Com_143	Shikimic acid	1.120717962	down
Com_15	3-(4-Hydroxyphenyl)propionic acid	2.258675525	up
Com_152	Serine 1	1.360572489	down
Com_158	Galactinol 1	1.510118139	up
Com_162	Linoleic acid	1.937025214	up

Com_165	1,3-Diaminopropane	1.12161045	up
Com_168	Citric acid	1.408479953	up
Com_171	Trehalose-6-phosphate	1.215633357	down
Com_172	Hesperitin 3	1.621660862	up
Com_183	Piceatannol 2	2.638671473	up
Com_189	Maltose	1.010534251	down
Com_192	Arachidic acid	1.152005298	up
Com_193	Aspartic acid 1	1.740163176	up
Com_194	D-erythronolactone 2	4.017246248	down
Com_195	Benzylamine	1.12045263	up
Com_197	Itaconic acid	1.332404611	up
Com_198	Glucose-6-phosphate 2	1.311751639	up
Com_20	Ribulose-5-phosphate 1	1.538219564	down
Com_200	N-Methyl-L-glutamic acid 1	1.270996219	up
Com_201	Galactose 2	1.604116553	up
Com_206	Alanine 1	1.163771181	down
Com_207	Atrazine-2-hydroxy 4	1.807306235	up
Com_209	Saccharic acid	2.688828454	down
Com_214	Methionine 1	1.015843381	down
Com_217	Epicatechin	2.857876857	up
Com_220	N-methylaniline	1.32253395	up
Com_225	lactic acid	2.906596568	down
Com_230	Gentiobiose 1	1.114514439	up
Com_231	Mucic acid	1.488639784	down
Com_24	N-Acetyl-D-galactosamine 1	1.23504606	up
Com_30	5-Methoxytryptamine 2	1.208451341	up
Com_34	2,4-diaminobutyric acid 1	1.18698222	up
Com_50	Threo-beta-hydroxyaspartate 1	1.537500053	up
Com_52	Salicin	1.159109938	up

Com_55	Ascorbate	1.457437314	up
Com_60	3-Hydroxynorvaline 1	1.329424699	up
Com_61	3-Hydroxybutyric acid	1.483762713	down
Com_63	(+)-Catechin	2.174593582	up
Com_65	Methyl Phosphate	2.673218786	up
Com_70	3-Hydroxypropionic acid 1	1.453604877	down
Com_73	Maltitol	1.313798378	up
Com_80	4-Hydroxybutyrate	2.157589383	down
Com_82	Fucose 1	1.348500127	down
Com_83	Nicotinamide	1.75663965	up
Com_85	Pyruvic acid	2.158038298	down
Com_89	trans,trans-Muconic acid	1.457448479	up
Com_90	Guanidinosuccinic acid 3	1.330197978	down
Com_93	Succinic acid	1.470168758	down
Com_96	Isomaltose 1	1.224279483	up

Table S3. The identified differential metabolites (VIP＞1) between group B vs group C. B-Tartary buckwheat in Sichuan, C-Tartary buckwheat in Yunnan.

ID	Name_des	VIP	Up.Down
Com_108	Myo-inositol	1.37259832	up
Com_115	Guanosine	1.600957769	up
Com_118	Proline	1.160077357	up
Com_119	3,6-Anhydro-d-galactose 3	1.270081806	up
Com_121	Glycine 1	1.246945821	up
Com_127	Adenine	1.491408774	up
Com_130	Glucoheptonic acid 1	1.404208015	up
Com_132	Lyxose 2	1.687664124	up
Com_135	Isoleucine	1.107405192	up
Com_141	4-Acetylbutyric acid 2	1.364632487	up

Com_146	Phytosphingosine 2	1.035434514	up
Com_164	Phenylalanine 1	1.011406996	up
Com_173	4-Aminobutyric acid 1	1.919484903	up
Com_181	Valine	1.132570652	up
Com_186	5-Aminovaleric acid lactam	1.950214271	up
Com_188	L-allothreonine 1	1.042464495	up
Com_19	Stearic acid	1.192944511	up
Com_191	Glycerol	1.300521121	up
Com_192	Arachidic acid	1.697918297	up
Com_194	D-erythronolactone 2	1.943446169	up
Com_2	D-erythro-sphingosine 2	1.762609211	up
Com_202	Canavanine 1	1.110204641	up
Com_203	D-talose 1	1.167688831	up
Com_204	D-talose 2	1.327206676	up
Com_205	Tyrosine 1	1.376271014	up
Com_214	Methionine 1	1.638754253	up
Com_225	Lactic acid	2.087175598	up
Com_23	N-methyltryptophan	1.05758331	up
Com_232	Palmitic acid	1.196873132	up
Com_30	5-Methoxytryptamine 2	1.038157769	up
Com_37	Ornithine	1.45748559	up
Com_40	(2r)-2-Amino-3-phosphonopropanoic acid 2	1.870037247	down
Com_51	Lysine	1.579184738	up
Com_56	Uracil	1.346756597	up
Com_66	Phenyl beta-d-glucopyranoside	1.552053463	up
Com_77	Cholestane-3,5,6-triol	1.714579014	up
Com_97	Xanthine	2.19747498	up
